# Long Amplicon Nanopore Sequencing for Dual-Typing *RdRp* and *VP1* Genes of Norovirus Genogroups I and II in Wastewater

**DOI:** 10.1007/s12560-024-09611-5

**Published:** 2024-09-06

**Authors:** G. Scott, D. Ryder, M. Buckley, R. Hill, S. Treagus, T. Stapleton, D. I. Walker, J. Lowther, F. M. Batista

**Affiliations:** 1 Centre for Environment, Fisheries and Aquaculture Science, Weymouth, UK; 2 UK Health Security Agency, Salisbury, UK

**Keywords:** Norovirus, Wastewater, Epidemiology, Environmental water, Sequencing, Nanopore

## Abstract

Noroviruses (NoVs) are the leading cause of non-bacterial gastroenteritis with societal costs of US$60.3 billion per annum. Development of a long amplicon nanopore-based method for dual-typing the *RNA-dependent RNA polymerase* (*RdRp*) and major structural protein (*VP1*) regions from a single RNA fragment could improve existing norovirus typing methods. Application to wastewater-based epidemiology (WBE) and environmental testing could enable the discovery of novel types and improve outbreak tracking and source apportionment. Here, we have developed such a method with a consensus-based bioinformatics pipeline and optimised reverse transcription (RT) and PCR procedures. Inhibitor removal and LunaScript® RT gave robust amplification of the ≈ 1000 bp *RdRP* + *VP1* amplicon for both the GI and GII PCR assays. Platinum™ Taq polymerase showed good sensitivity and reduced levels non-specific amplification (NSA) when compared to other polymerases. Optimised PCR annealing temperatures significantly reduced NSA (51.3 and 42.4% for GI and GII), increased yield (86.5% for GII) and increased taxa richness (57.7%) for GII. Analysis of three NoV positive faecal samples showed 100% nucleotide similarity with Sanger sequencing. Eight GI genotypes, 11 polymerase types (p-types) and 13 combinations were detected in wastewater along with 4 GII genotypes, 4 p-types and 8 combinations; highlighting the diversity of norovirus taxa present in wastewater in England. The most common genotypes detected in clinical samples were all detected in wastewater while we also frequently detected several GI genotypes not reported in the clinical data. Application of this method into a WBE scheme, therefore, may allow for more accurate measurement of norovirus diversity within the population.

**Supplementary Information:**

The online version contains supplementary material available at 10.1007/s12560-024-09611-5.

## Introduction

Noroviruses (NoVs) are non-enveloped viruses in the family *Caliciviridae* with a single stranded, positive sense RNA genome. In human NoV strains the genome is ≈ 7.6 kb in length and comprised of three open reading frames (ORFs). ORF-1 encodes the non-structural proteins P48, NTPase, P22, VPg, Protease and RNA-dependent RNA polymerase (RdRp) while ORF-2 and -3 encodes the major structural capsid protein VP1 and minor structural protein VP2 (Campillay-Véliz et al., 2020). Five of the ten NoV genogroups (GI, GII, GIV, GVII and GIX) infect humans, although the majority of reported NoV cases are GI and GII (Chhabra et al., 2019; Ham et al., 2015). Further classification into genotypes and polymerase-types (p-type) is based on the *VP1* and *RdRp* regions*,* with a dual-typing system proposed by Chhabra et al. (2019) naming of both the genotype (e.g. GI.2) and p-type (e.g. GI.P2) in combination (e.g. GI.2[P2]); referred to herein as type.

The leading cause of epidemic and non-bacterial gastroenteritis, NoV was estimated to cost US$4.2 billion in direct health costs and US$60.3 billion in societal costs worldwide in 2016 (Baldridge et al., 2016; Bartsch et al., 2016; Campillay-Véliz et al., 2020). Although severe disease and death is rare, mortality rates are higher in children < 5 y old, the elderly and immunocompromised (Baldridge et al., 2016; Bartsch et al., 2016). Out of an estimated 699 million illnesses, 219,000 deaths are predicted to occur annually; 70,000 being children < 5 y old (Baldridge et al., 2016; Bartsch et al., 2016). The main transmission route is faecal-oral and an acute symptomatic phase of 1–4 d occurs with faecal shedding commencing at 0.8 d which may continue for months (Baldridge et al., 2016; Ge et al., 2023). High viral loads (~ 2.0 × 10^9^ genome copies/g stool) and particle stability make NoV a suitable candidate for wastewater based epidemiology (WBE) (Hall, 2012; Newman et al., 2016).

WBE became prominent during the SARS-CoV-2 pandemic with at least 72 countries adopting it to quantify viral RNA, track variants of concern and emerging variants (Naughton et al., 2021). WBE, however, has been used for decades as part of the poliovirus eradication programme and for monitoring other pathogens such as hepatitis viruses and norovirus (Asghar et al., 2014; Polo et al., 2020). It has the potential to be a useful tool with benefits including detection of asymptomatic cases, access to near population-scale epidemiological information without mass testing and fewer anthropogenic biases.

WBE studies have already shown that NoV quantities in wastewater can be predictive (2–4 weeks prior) or are coincident with clinical cases (Boehm et al., 2023; Hellmér et al., 2014; Kazama et al., 2017; Markt et al., 2023). Metagenomic and amplicon sequencing approaches have been used to type NoVs. Strubbia et al. (2019) detected six types GI.4[P4], GI.2[P2], GI.1[P1], GII.6[P7] GII.17[P17] and GII.4 Sydney [P16] from three composite samples from a city with population of ≈ 0.3 million using a metagenomic approach paired using MiSeq 2 × 150 bp (Illumina, USA). Six full-length genomes (> 7000 nt) were recovered highlighting the benefit of a metagenomic approach which will not be affected by PCR bias but may suffer from reduced sensitivity.

Numerous studies using amplicon sequencing to genotype and p-type NoV in wastewater have been performed. The majority amplify partial *VP1* or *RdRp* regions in isolation. Kazama et al. (2017) and Cao et al. (2022) used semi-nested PCR of partial GI and GII *VP1* regions (≈ 337 bp) using the GS-Junior (Roche, Switzerland) and NovaSeq (Illumina, USA) platforms. In both cases 15 genotypes were detected, with up to 8 genotypes per sample for the former. Fumian et al. (2019) amplified the 5ʹ of the GII *VP1* (373 bp) using MiSeq 2 × 150 bp (Illumina, USA). Over 1 yr, 13 genotypes were detected from 156 samples with GII.4 being the most prevalent. Mabasa et al. (2022) sequenced ≈ 575 bp of the ORF-1 and -2 junction using MiSeq 2 × 300 bp (Illumina, USA). Over 26 months of bi-weekly sampling, 81% were positive and 13 and 21 types of GI and GII were detected. Recently our group used nanopore sequencing to genotype GII (302 bp amplicon) and found 8 genotypes across 42 samples (Treagus et al., 2023). These previous studies highlight the diverse range of NoV types that can be detected in wastewater.

Most previous research, however, has relied on short-read sequencing which often require separate amplification and sequencing of *VP1* and *RdRp.* This makes application of dual-typing post-sequencing extremely difficult due to high levels of recombination in the norovirus genome. The aim of this study, therefore, was to develop a long-amplicon (≈ 1000 bp) nanopore sequencing method optimised for wastewater that allows dual-typing of GI and GII. Development of such an approach could allow identification of emerging variants and novel recombinants from wastewater.

## Materials and Methods

A step-by-step protocol for the methods developed in this study is available in Online Resource 2 or on protocols.io at 10.17504/protocols.io.8epv5xpmjg1b/v2.

### Sample Collection and Processing

Samples were collected and processed by the Environment Agency as part of the SARS-CoV-2 wastewater monitoring programme in England (UK) as outlined in Walker, (2024). Briefly, 1 L of untreated sewage was collected and processed (150 mL) using ammonium sulphate precipitation and nucleic acid extraction using a Kingfisher Flex™ (Thermo Scientific™, UK) and NucliSENS® (BioMérieux, France) reagents. Nucleic acids were sent on dry ice to the Centre for Environment, Fisheries and Aquaculture Science (Weymouth, UK).

To assess the performance of inhibitor removal and the wastewater optimised sequencing protocol, 209 nucleic acid extracts from wastewater collected between 22/10/2021 and 25/10/2021 were pooled by geographical region based on the location of the wastewater treatment plants using QGIS 3.16 and the “Regions (December 2021) EN BFC” (Office for National Statistics, 2021; QGIS Development Team, 2021). Regions with limited or excess nucleic acid volume were split, combined or omitted creating 10 samples (Online Resource 1, Supplementary Table 1). Excess nucleic acids were pooled into three additional independent pools and used to assess reverse transcription, PCR and size selection.

### Inhibitor Removal

To assess the impact of inhibitors on reverse transcription and PCR, nucleic acid extracts were cleaned with Mag-Bind® TotalPure NGS beads (Omega Bio-Tek, USA) following Child et al. (2023) using 25 µL of nucleic acids. To monitor inhibitor removal efficacy, a GI RT-qPCR and an external control RNA (EC RNA) method was used following ISO 15216-1: 2017 (ISO, 2017). Reactions were spiked with 1 µL of EC RNA (4000 genome copies/µL) and run alongside an EC RNA + water control. Reactions were run in duplicate on QuantStudio™ 3 machines. RT-qPCR standard curve slopes were between − 3.6 and − 3.1 with R^2^ ≥ 0.99. Technical repeats were averaged prior to analysis. Inhibition values < 0% were assigned a value of 0 and NoV concentration data was square root transformed (West, 2022).

### Reverse Transcription Optimisation

Methods using two reverse transcription (RT) kits were tested; SuperScript™ IV Reverse Transcriptase (Invitrogen™, USA) and LunaScript® RT SuperMix (New England Biolabs, USA), referred to as Superscript™ and LunaScript®. For SuperScript™, the manufacturer’s instructions were followed for a 20 µL final volume, 10 µL of nucleic acids and RT at 50 °C. LunaScript® followed Child et al. (2023) with a nucleic acid volume of 10 µL with 7.5 µL of molecular biology grade water added after RT to equalise sample dilution.

To assess RT performance, semi-nested PCR of the *RdRp* + *VP1* region was performed. First round and semi-nested products were 1194 and 1110 bp for GI and 1052 and 971 bp for GII, respectively (Table 1). PCR with Platinum™ Taq DNA Polymerase followed the manufacturer’s instructions for a 25 μl reaction with 5 μl of cDNA or first-round PCR products. Cycling conditions were 95 °C for 1 min followed by 40 cycles of 95 °C for 30 s, 50 °C for 30 s and 72 °C for 30 s and 72 °C for 7 min. Products were visualised by gel electrophoresis with 2% tris–borate EDTA agarose (Sigma Aldrich) with 100 bp ladder (Promega, USA) or with a TapeStation 4150 using D5000 screen tape (Agilent, USA).

**Table 1:** Primers used for amplification of the RdRp + VP1 region

Name	Genogroup	F/R	Sequence (5ʹ–3ʹ)	References
NV4478_m_	GI	F_1_	AA**RY**T**V**CCHATHAA**R**GTTGGNATG	Ollivier et al. (2022)
NV4562	GI	F_2_	GATGCDGAYTAYACRGCHTGGG	Yuen et al. (2001)
GISKR_m_	GI	R_1,2_	CC**I**ACCCA**I**CCATTRTACA	Yuen et al. (2001)
NV4611	GII	F_1_	CWGCAGCMCTDGAAATCATGG	Yuen et al. (2001)
NV4692	GII	F_2_	GTGTGRTKGATGTGGGTGACTT	Yuen et al. (2001)
GIISKR	GII	R_1,2_	CCRCCNGCATRHCCRTTRTACAT	Kojima et al. (2002)

Subscript represent (1) first round primers and (2) semi-nested primers and (m) original primer modifications. Bold letters indicate primer modifications

### PCR Optimisation and Size Selection

Two independent pooled cDNA samples processed using LunaScript™ were used to optimise PCR. Six polymerases were assessed; LongAmp® Hot Start (New England Biolabs, USA); Phusion™ Hot Start II DNA Polymerase (Invitrogen™, USA); Platinum™ Taq polymerase (Invitrogen™, USA); Q5U® Hot Start High-Fidelity DNA Polymerase (New England Biolabs, USA); NEB Next® Ultra™ II Q5® mastermix (New England Biolabs, USA); and Platinum™ SuperFi™ DNA Polymerase (Invitrogen™, USA) referred to herein as LongAmp®, Phusion™, Platinum®, Q5U®, Ultra™ II Q5 and SuperFi™.

Annealing temperatures (T_a_) were optimised using gradient PCR with the lowest primer melting temperature (T_m_) for each polymerase as the highest T_a_ with two additional temperatures at ≈ 2.5 °C and ≈ 5.0 °C below. Cycling conditions were as recommended by the manufacturer and run for 40 cycles. Reactions were run in simplex at 1 and tenfold dilutions on a Mastercycler® Nexus Gradient (Eppendorf, Germany). Optimised PCR T_a_ were compared to T_a_ = 50 °C as used by Ollivier et al. (2022). Ampure XP (Beckman Coulter, USA) and Mag-Bind® TotalPure NGS (Omega Bio-Tek, USA) beads were trialled for size selection using both 0.4–0.6× ratios based on the manufacturers’ recommendations.

### Library Preparation and Sequencing

To simultaneously type GI and GII under a single barcode, an amplicon pooling method was developed to increase the equality of sequencing depth. Nucleic acid extracts (158) from untreated sewage samples collected between 05/08/21 and 11/08/21 and 25/02/22 and 07/03/2022 were processed using the wastewater optimised methods and then analysed by TapeStation. The amplicon of interest percentage AOI% was calculated following Online Resource 1, Supplementary Eq. 1. Negative and diluted samples, those with unusually high levels of non-specific amplification (NSA) and outliers (1.5 times the interquartile range) were removed and the average GI:GII AOI% ratio was determined (Online Resource 1, Supplementary Eq. 2). This was used to adjust the moles of the GI and GII PCR products input into library preparation (Online Resource 1, Supplementary Eqs. 3 and 4).

PCR products were purified using ExoSAP-IT™ (Applied Biosystems, USA) following the manufacturer’s instructions for 10 µL of product. PCR yield was determined using a Qubit™ Flex (Invitrogen™, USA) and dsDNA High Sensitivity kit with 2 µL of sample. For the wastewater optimised assay, 85.3 and 114.7 fmol of GI and GII amplicons were pooled together. For the unoptimised method, this was 62.0 and 138.0 fmol of GI and GII. These adjustments were based on the GI:GII AOI% ratios of the methods. Library preparation was performed using the Native Barcoding Kit 96 V14 (Oxford Nanopore Technologies, UK) following the manufacturer’s instructions for sequencing of amplicons. Sequencing was performed on a GridION (MinKNOW software release 22.10.7) using R10.4.1 flow cells at 260 bps with super accurate basecalling. Whole process (cDNA synthesis onwards) and PCR-specific no template controls were sequenced alongside the samples.

### Bioinformatics

Briefly, reads were split using duplex_tools 0.2.14 and trimmed with cutadapt 3.4 (Martin, 2011; Oxford Nanopore Technologies, 2022). Size filtering and random sampling (> 800 bp and ≤ 90,000 reads per sample) was performed using SeqTK 1.3 (Li, 2018b). Minimap 2.24 was used to find overlaps between reads. Alignments were screened using yacrd 1.0.0 to detect chimeras or poorly supported reads where more than 20% of a given read had a depth of less than 10 (Li, 2018a; Marijon et al., 2020). NGSpeciesID 0.1.3 clustered reads and formed consensus sequences supported by more than 100 reads (Sahlin et al., 2021).

For each sample, consensus sequences were indexed, polished and screened for regions with poor support and reads aligned using kma 1.4.9 (Clausen et al., 2018). All consensus sequences were concatenated, renamed using SeqTK 1.3 and then indexed with Samtools 1.13 (Danecek et al., 2021; Li, 2018b). SeqKit 2.3.0 identified regions at consensus termini soft masked by kma 1.4.9 due to having poor support, with any such regions being trimmed using Bedtools 2.30.0 (Quinlan & Hall, 2010; Shen et al., 2016). Consensuses were clustered at 95% (CD-HIT 4.8.1) and typed using the Centers for Disease Control and Prevention’s (CDC) Human Calicivirus Typing Tool (Fu et al., 2012; Tatusov et al., 2021). Full bioinformatic procedures, tools and commands can be found in Online Resource 1, Supplementary Table 2 or on protocols.io 10.17504/protocols.io.8epv5xpmjg1b/v2.

Untyped consensus sequences were aligned against CDC reference sequences in MEGA 11.0.11 using the ClustalW algorithm with default parameters (Centers for Disease Control & Prevention, 2023; Tamura et al., 2021). Indels causing frame-shift mutations were assessed using amino acid alignments and putative viral typing using the CDC’s typing tool (as above) followed by error correction. R 4.1.2 was used to collate the number of reads mapped to each of the consensus sequences (R Core Team, 2021).

Putative PCR chimeras were removed from the dataset if they met any of the following criteria: Failing to align against the CDC’s reference sequence database (Centers for Disease Control & Prevention, 2023), identification as a chimera by USEARCH v11 de-novo or a child sequence with a parent breakpoint within the terminal or proximal regions of *RdRp* and *VP1* and child-parent sequence similarities ≥ 95%. For USEARCH chimera detection, consensus sequences were annotated with read count and screened using USEARCH v11 de-novo chimera detection (Edgar, 2016). Manually screening was performed using NCBI Multiple Sequence Alignment Viewer v1.25.0. For the method comparison study, a read-depth threshold of 0.1% of the median reads per sample was used and reads from consensus sequences of the same viral type were grouped.

Nanopore-generated sequences were assessed against those obtained by Sanger sequencing. Three norovirus positive faecal samples as determined by RT-qPCR following ISO 15216-1:2017 from presumed single-type infections (ISO, 2017), were processed following the wastewater optimised protocol. PCR products were purified using the QIAquick PCR Purification Kit (QIAGEN, Germany) and sequenced with the forward primer using Mix2Seq (Eurofins, Germany). Sequences were trimmed to 10 consecutive Q30 bases prior to alignment. The above bioinformatic approach was used without consensus sequence clustering at 95%. Read quality was estimated by aligning reads against the faecal samples consensus sequences using minimap 2.26 and the map-ont preset. Results were filtered to include only those alignments ≥ 700 bp. Quality scores were calculated as described in Online Resource 1, Supplementary Eq. 5.

### Data Analysis

For all inferential statistics, paired t-tests were performed unless the assumption of normality of the difference between observations was not met where a Wilcoxon signed-rank test was performed. All data analysis and visualisation was performed in R Studio build 353 (2022.12.0) and statistical significance is defined as p < 0.05 (Posit team, 2022).

## Results

### Inhibitor Removal and Reverse Transcription

**Fig. 1:**
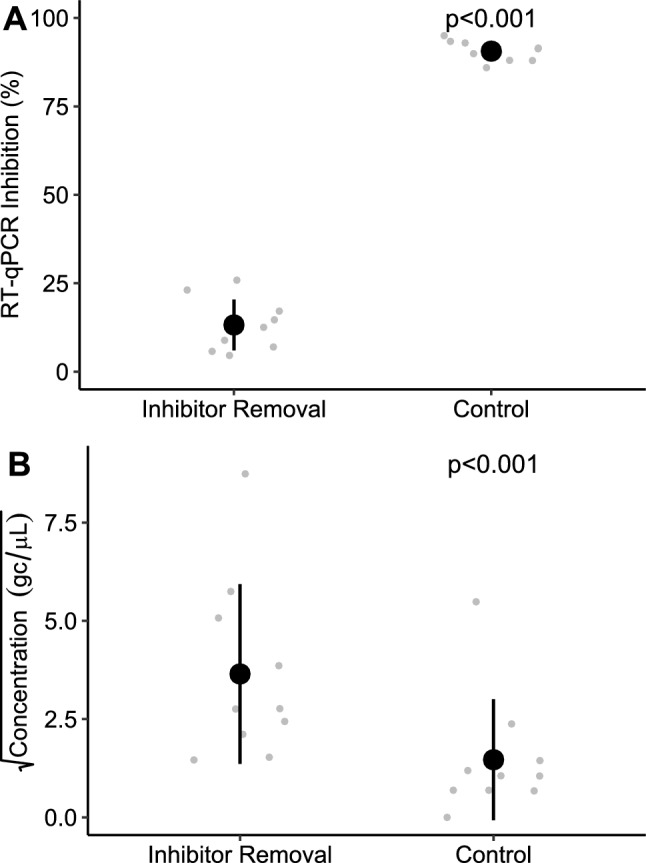
The RT-qPCR inhibition (**A**) and concentration (**B**) of norovirus GI from cDNA samples processed with or without inhibitor removal where gc = genome copies. Black markers show the average and grey show the raw data; error bars show ± 1 sd. N = 10

**Fig. 2:**
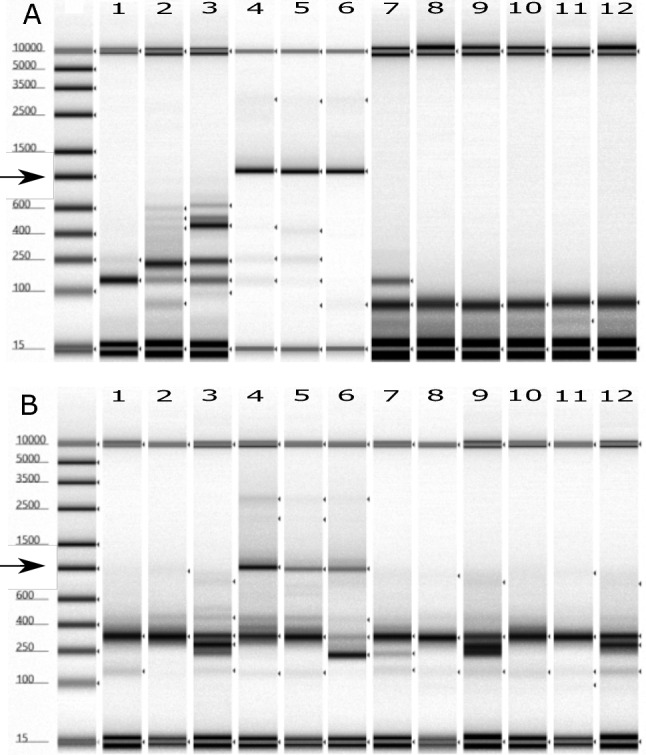
TapeStation images showing the RdRp + VP1 amplicon for GI (**A**) and GII (**B**) using two different reverse transcriptases, LunaScript® (1–6) and SuperScript™ (7–12). Samples were processed without (1–3 and 7–9) or with (4–6 and 10–12) inhibitor removal. Products are 1110 and 971 bp for GI and GII. Black arrow indicates the expected amplicon size, n = 3

Inhibitor removal significantly reduced average inhibition from 90.6% to 13.2% and increased NoV quantification 4.8-fold; p < 0.001 (Fig. 1A, B). Reverse transcription studies showed no or weak amplification with SuperScript™ while Lunascript® showed good amplification for GI and GII with inhibitor removal (Fig. 2A, B). For Lunascript®, the number of aligned reads ≥ 900 bp (log_10_) significantly increased with inhibitor removal from 1.99 ± 0.25 (sd) to 5.22 ± 0.24 for GI and 2.31 ± 0.38 to 4.52 ± 0.46 for GII (p < 0.001) giving 163-fold and 1731-fold differences.

### PCR Reagents, Cycle Optimisation and Amplicon Purification

**Fig. 3:**
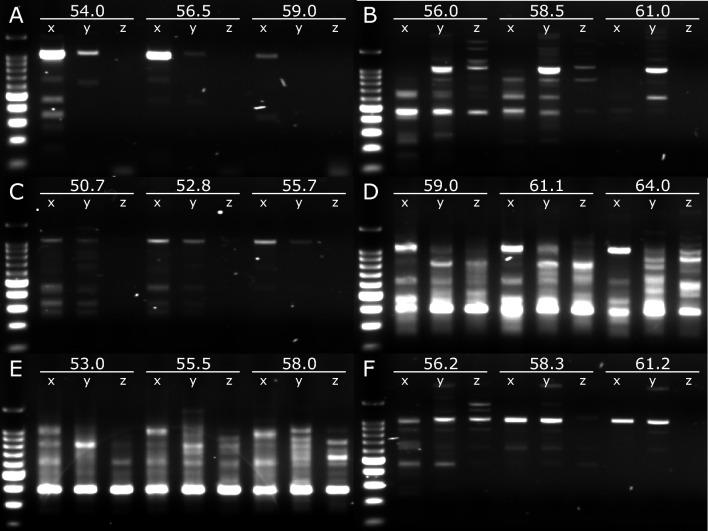
Gel electrophoresis of the first-round GII PCR products from several polymerases **A** LongAmp®, **B** Phusion™, **C** Platinum™, **D** Q5U®, **E** Ultra™ II Q5® and **F** SuperFi™. Numbers indicate the annealing temperature (°C) and x, y, and z are 1-, 10- and 100-fold diluted cDNA. Expected PCR products are ~ 1 kb (second marker) of the 100 bp DNA ladder (Promega, USA). N = 2, second sample not shown

Following first round PCR, no GI amplicons were observed indicating that the polymerases lacked the sensitivity to amplify NoV GI in a single round. For GII, however, amplicons were observed but not for all dilutions (Fig. 3). Phusion™ showed signs of PCR inhibition with increased yields following dilution (Fig. 3B). Q5U® and Ultra™ II Q5® showed abundant NSA at all T_a_ (Fig. 3D, E). In general, Platinum™ and LongAmp® showed low levels of NSA, good yield and sensitivity with both showing amplification of samples diluted tenfold at their optimal T_a_. For LongAmp™, yield reduced with increasing T_a_ but Platinum™ increased with T_a_ while SuperFi™ showed a reduction in sensitivity and NSA but an increase in yield with increasing T_a_ (Fig. 3A, C, F).

**Fig. 4:**
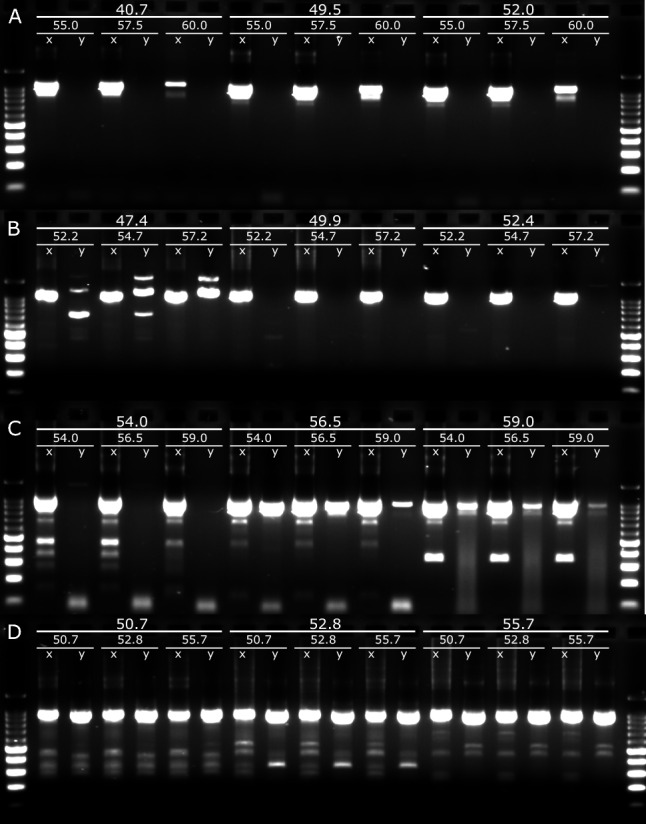
Gel electrophoresis of the semi-nested PCR products of GI (**A**, **B**) and GII (**C**, **D**) using polymerases LongAmp® (**A**, **C**) and Platinum™ (**B**, **D**). Numbers indicate first-round and semi-nested annealing temperatures. Samples were run at 1 (**x**) and 100-fold (**y**) dilution. Expected PCR products are ~ 1 kb (second marker) on the 100 bp DNA ladder (Promega, USA). N = 2, second sample not shown

For the semi-nested PCR, LongAmp® and Platinum™ were pursued as SuperFi™ cannot process the inosine bases present in GISKR_m_. Platinum™ showed the best sensitivity for GI with amplification at 100-fold dilution and lower NSA compared to LongAmp® (Fig. 4A, B). Optimum T_a_ was 47.4 and 57.2 °C for the first-round and semi-nested PCR, respectively (Fig. 4A). For GII, Platinum™ showed better sensitivity compared to LongAmp® and its NSA was slightly reduced at T_a_ = 55.7 °C for first-round and semi-nested PCR (Fig. 4C, D).

### Development of a Multi-Target Library

**Fig. 5:**
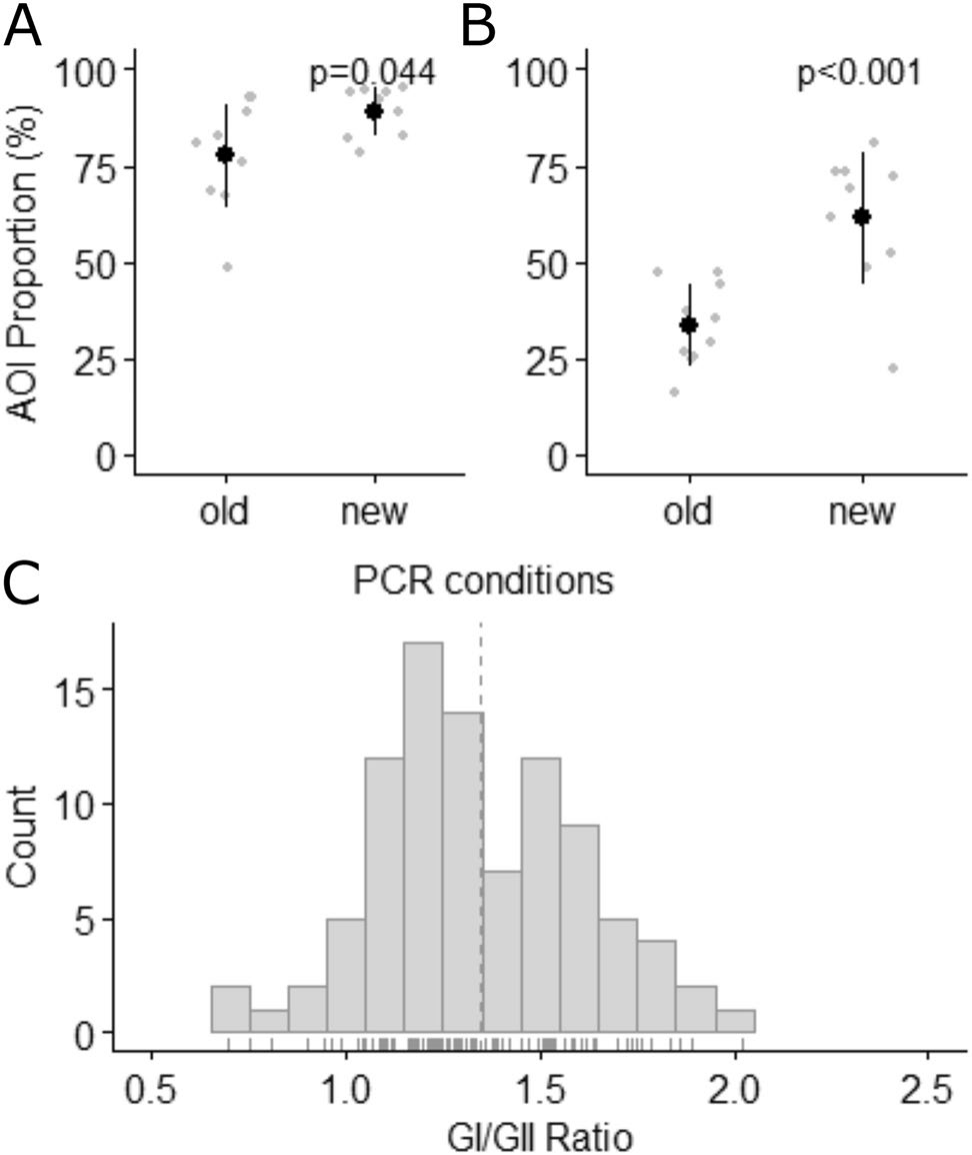
The percentage of the amplicon of interest (AOI) within the total amplicon pool for the T_a_ = 50 °C (old) and optimised (new) PCRs for norovirus GI (**A**) and GII (**B**). The GI/GII AOI ratios (**C**). For **A** and **B** n = 10, the black dots show the average AOI% and grey dots show individual sample data. Error bars show ± 1 sd. For **C**, n = 130 and dashed line indicates the mean

Following PCR optimisation, NSA was higher for GII than GI with the AOI% at 61.5 and 89.1% (Figs. 4B, D and 5A, B). PCR optimisation significantly increased AOI% for GI and GII from 77.6 to 89.1% and 33.4 to 61.5% (Fig. 5A, B). PCR yield significantly increased for GII (p < 0.001) following optimisation from 11.84 ng/µL ± 2.71(sd) to 22.08 ng/µL ± 3.47 while GI was not impacted; 29.97 ng/µL ± 4.47 and 29.78 ng/µL ± 2.80.

To reduce sequencing of NSA, Ampure XP- and Mag-Bind® TotalPure NGS-based size selection methods were trialled. Neither increased the AOI% (78.4–79.4%) but both saw reductions in amplicon concentrations (24.1–6.14 ng/µL); highlighted in Supplementary Fig. 2A, B. A library pooling method based on the AOI% within the total amplicon pools for GI and GII was developed with a 1.34-fold mean difference between the GI and GII AOI% being identified (Fig. 5C). Adjusting the GI and GII pooling molarities reduced the bias in median read coverage from 3.3-fold to 2.6-fold.

### Sequence Quality, Nanopore Sequence Validation and PCR Chimeras

During comparative analysis of the wastewater optimised methods and those using T_a_ = 50 °C, whole process and PCR negative controls were free from any sequences aligning to NoVs (Online Resource 1, Supplementary Table 9). Prior to read trimming the mean and median read lengths were 1790 and 851 bp, respectively, suggesting a positively skewed distribution with a few very long reads. Approximately 45.5–71.2% of reads per sample included both primers and of those 17.2–45.2% aligned against a consensus sequence (Online Resource 1, Supplementary Table 9). Of 94 consensus sequences, 3 couldn’t be typed due to indels in homopolymer regions.

To validate the nanopore consensus sequences, three faecal samples from assumed single-type infections were compared to sequences obtained by Sanger sequencing. All three had 100% nucleotide similarity compared to Sanger with no indels. The Sanger sequences had median and modal quality scores of Q58 (Online Resource 1, Supplementary Table 3). Alignment of nanopore reads from these samples against the consensus sequences estimated the median quality of reads in the sequencing library as being 12.3 (Online Resource 1, Supplementary Fig. 1).

During initial data processing, 12 putative novel recombinant GI types were observed on 18 occasions (Online Resource 1, Supplementary Table 4). On 16 occasions, parent-types containing the genotype or p-type were both present at ≥ 7.4% of the total reads with the recombinants comprising ≤ 2.5%; indicating their potential as PCR chimeras. On two occasions the parent types (GI.1[P1] and GI.2[P2]) of the putative chimeras (GI.1[P2] and GI.2[P1]) were not detected in the sample. Some putative chimeras contained a low number of SNPs (< 5) compared to their parent types.

### The Impact of PCR Optimisation on Norovirus Diversity

**Fig. 6:**
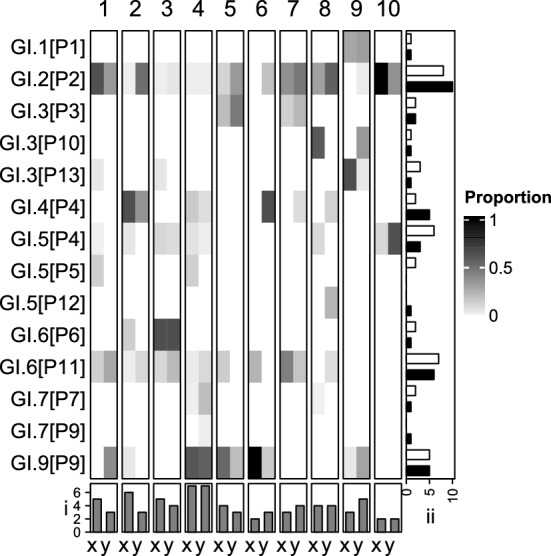
Norovirus genogroup I types identified in ten pooled wastewater samples (1–10) using two PCR assays (x and y). Assay x used T_a_ = 50 °C while y is the wastewater optimised assay. The heatmap shows the proportion of reads assigned to each of the type. (**i**) is taxa richness and (**ii**) is the frequency of observations for the two different methods x (white) and y (black)

**Fig. 7:**
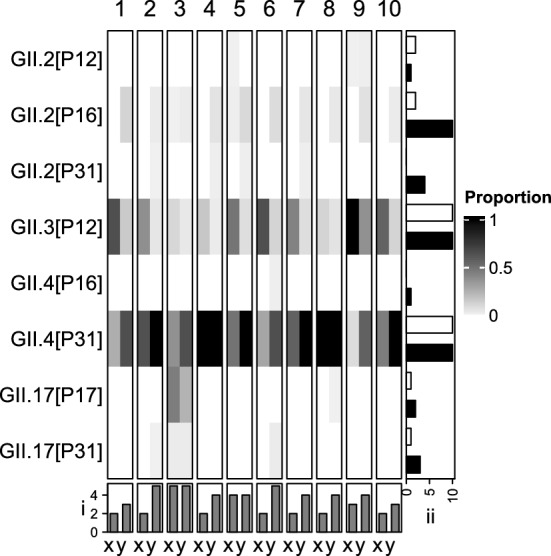
Norovirus genogroup II types identified in ten pooled wastewater samples (1–10) using two PCR assays (x and y). Assay x used T_a_ = 50 °C while y is the wastewater optimised assay. The heatmap shows the proportion of reads assigned to each of the type. (**i**) is taxa richness and (**ii**) is the frequency of observations for the two different methods x (white) and y (black)

Ten pooled wastewater samples were analysed to determine the impact of PCR optimisation on NoV taxa richness. The optimised PCR detected 13 GI types, two (GI.5[P12] and GI.7[P9]) were not detected by the T_a_ = 50 °C method. Following optimisation, taxa observations reduced from 41 to 38 along with taxa richness from 4.1 ± 1.7 to 3.8 ± 1.4 (p = 0.729) (Fig. 6i, ii). Differences in detected taxa were from types whose genotypes and p-types had been detected separately in different samples; apart from GI.P5 which was not detected by the optimised assay. For GII, the total number of types detected increased from 6 to 8 after optimisation with T_a_ = 50 °C not detecting GII.2[P31] or GII.4[P16] (Fig. 7). Total taxa count increased from 26 to 41 and taxa richness significantly increased from 2.6 ± 1.07 and 4.1 ± 0.7 (p < 0.001) (Fig. 7B i and ii).

### Noroviruses in Wastewater in England

This section reports the data from the wastewater optimised assay. Aside from GI.8, all GI genotypes and 11 out of 14 GI p-types described by the CDC were detected from the pooled samples collected from across England (Online Resource 1, Supplementary Tables 5 and 6) (Centers for Disease Control & Prevention, 2023). Analysis of the genotype:p-type combinations identified up to 3 p-types per genotype and two genotypes per p-type, whilst both had a modal number of 1 pairing (Fig. 6A). Four taxa GI.2[P2], GI.4[P4], GI.6[P11] and GI.9[P9] occurred in ≥ 50% of the samples (100, 50, 60 and 50%) with GI.2[P2] and GI.9[P9] tending to be the dominant taxa when present (Fig. 6).

Eight GII types, representing 4 of the 26 genotypes and 4 of the of the 37 p-types as described by the CDC were detected (Online Resource 1, Supplementary Tables 7 and 8) (Centers for Disease Control & Prevention, 2023). Up to 3 p-types per genotype and 3 genotypes per p-type were observed with a mode of 2 for both. Three GII-types (GII.2[P16], GII.3[P12] and GII.4[P31]) were detected in 100% of the samples with GII.4[P31] having the highest proportion of reads in all cases (Fig. 7).

## Discussion

Two of the challenges faced during method development were the physiochemical and microbiological nature of wastewater, their impact on NoV detection and the biology of NoVs themselves. As enteric viruses, NoVs are detected frequently (82–100%) in wastewater making NoV negative samples difficult to obtain (Huang et al., 2022; Qiu et al., 2021). Performing method validation with spiked-matrix mock communities is, therefore, difficult to achieve. Additionally, difficulties obtaining clinical samples and culturing NoVs makes obtaining a diverse range of reference material for artificial matrix-based experimentation challenging. Furthermore, such experiments fail to account for the potentially large matrix-effect that wastewater exhibits (Scott et al., 2023). This creates issues around determination of PCR chimeras, quantitative validation and calculating sequencing error rates.

### Wastewater and RT-PCR Inhibition

Wastewater’s physicochemical properties are poorly characterised and are likely influenced by many geospatial and environmental factors. RT and PCR inhibitors likely present include polysaccharides, bile salts, lipid, urate, fulvic and humic acids, metal ions, algae and polyphenols (Schrader et al., 2012; Sims & Kasprzyk-Hordern, 2020). Inter-sample RT-qPCR inhibition levels have been shown to be highly variable (0–98%) for SARS-CoV-2 (Scott et al., 2023). Inhibition is, therefore, likely to influence the data quality of PCR-dependent WBE methods.

Here, we confirm the suitability of Child et al. (2023)’s inhibitor removal method for detecting NoVs in wastewater, reducing inhibition (85%) and increasing NoV quantification (4.8-fold) as measured by RT-qPCR (Fig. 1A, B). These data should be used with caution as inhibitor susceptibility may change between different enzymes and amplicons; quantitative performance gains for this metabarcoding approach cannot be inferred (Huggett et al., 2008; Kermekchiev et al., 2009). Following inhibitor removal, LunaScript™ showed 163- to 1731-fold increases in sequencing depth highlighting the large impact inhibitors can have on amplicon sequencing and the importance of matrix-specific assay optimisation.

### Wastewater and PCR Optimisation

The importance of matrix-specific assay optimisation is emphasised by wastewater’s likely diverse nucleic acid content with influence from humans, their food, microbial ecology of the gastrointestinal tract and other natural and industrial sources of wastewater. This makes designing nucleic acid-based assays challenging due to an increased likelihood of NSA. Furthermore, nucleic acid degeneracy within primers is required to capture the full genomic diversity of NoVs; again increasing the likelihood of NSA.

Several polymerases were assessed for sensitivity, NSA and yield. Platinum™ showed the best performance overall for both GI and GII PCR assays (Figs. 3 and 4). It is likely that Platinum™ shows increased enzymatic activity at higher temperatures allowing good performance at higher T_a_ thereby reducing NSA and increasing yields. Optimisation of the Platinum™ PCR conditions significantly increased taxa richness for GII (57.7%) while a non-significant reduction was seen for GI. Assay optimisation increased the frequency of observation for some taxa; for example GII.2[P16] was detected in 20% of the samples using the T_a_ = 50 °C method but in 100% with the optimised protocol. Others (GII.2[P31] & GII.4[P16]) weren’t detected using the T_a_ = 50 °C method (Fig. 6B). This is likely due increased T_a_ reducing primer-template interactions when mismatches are present allowing the preferential amplification of NoV. Failure to properly optimise methods implemented in WBE is, therefore, likely to underestimate the diversity of the organisms under investigation.

Although PCRs were optimised to reduce NSA, a read-depth disparity for GI and GII remained. Size selection using magnetic beads to remove NSA was unsuccessful, perhaps because NSA were close in size to the AOI. As such, to reduce method complexity, PCR products were purified using ExoSAP-IT™. To account for the differences in depth, a target-specific weighting was then applied to the loading of the GI and GII amplicons into library prep.

### Sequencing and Bioinformatics

GI and GII were sequenced under a single barcode per sample to maximise throughput. Given the high estimated read error rate (median ≈ Q13) a consensus-based approach was used and analysis was focused at the type-level. Consensus sequences were clustered at 95% prior to read alignment as this threshold allowed for differentiation of types while accounting for noise in the data. GII sub-types, however, cannot be determined as thresholds are 98% (Tatusov et al., 2021). Consensus sequences used to identify novel recombinants and putative PCR chimers were supported by high-quality alignments of ≥ 900 bp in length, included ≥ 90% of a read and had normalised read scores of ≥ 92%.

A large amount of sequencing data were filtered prior to alignment, which appeared to be due to NSA and the high error rate inherent in nanopore sequencing. The latter should improve with new developments in nanopore sequencing chemistry, basecalling and flowcells. Despite most of the analysis focussing on types, analysis of faecal samples from three presumed single type NoV infections showed 100% nucleotide similarity with Sanger sequencing; with the latter having a median basecalling score of Q58 (Online Resource 1, Supplementary Table 3). This indicates that this method is adequate to discriminate between subtypes if the consensus sequence clustering is performed at 98% rather than the 95% implemented here.

### PCR Chimeras

Twelve putative novel GI *RdRp* + *VP1* recombinants were identified but removed as putative PCR chimeras. For most cases, putative chimeras were detected alongside parent types at higher abundance. On two occasions, only a single parent was identified, increasing the likelihood that the novel recombinant was genuine. Chimera formation without the detection of the parent, however, was observed by Ollivier et al. (2022).

Following artificial bioaccumulation of oysters with GII.4[P16], GII.2[P16], GII.4[P31] and GII.17[P17] the detection of two chimeras GII.17[P16] and GII.17[P13] occurred without detection of GII.17[P17]. This method used a shorter amplicon (426 bp) targeting the ORF-1 and -2 junction using Illumina MiSeq. Failure to detect both parent types may indicate forward primers failed to amplify GII.P17. In our study, however, the two parent types (GI.1[P1] and GI.2[P2]) of the putative chimeras (GI.1[P2] and GI.2[P1]) were detected in other samples (Fig. 6). This gives further indication that the novel recombinants may be genuine.

Both putative chimeras, however, formed a low proportion (0.4%) of the total reads (Online Resource 1, Supplementary Table 4). Due to wastewaters high probability of containing degraded nucleic acids, further investigation is required to determine whether these novel recombinants are genuine. Degradation of nucleic acids may prevent the detection of a parent type but still allow PCR chimera formation. This increases the likelihood of chimera formation from that of prematurely terminated strand elongation alone (Meyerhans et al., 1990). These putative chimeras were removed from the data due to the lack of additional evidence. Some putative chimeras contained up to 5 SNPs compared to parent types. Although SNP presence usually provides evidence against a PCR chimera, SNPs may be introduced during clustering of consensus sequences at 95% and as such were removed.

Differentiating between true recombinants and PCR chimeras *in-silico* is challenging. Methods using reference-based chimera filtering won’t detect novel recombinants or remove chimeras forming known types. De-novo approaches rely on read depth to detect chimeras and may miss chimeras with similar read depths to parents due to PCR bias. This is highlighted by the USEARCH de-novo approach missing several potential chimeras which were removed following manual screening. Additional investigation into the natural NoV recombination breakpoints did not assist in identification of chimeras due to its variability within the terminal and proximal ends of *RdRp* and *VP1* (Bull et al., 2005; Fu et al., 2019).

PCR chimera formation is not an issue isolated to long amplicon sequencing methods. Putative PCR chimeras were identified by Mabasa et al. (2022) who detected with 51 putative novel recombinants using a shorter amplicon (≈ 575 bp). PCR chimera formation cannot be discounted as their formation will be influenced by polymerase choice, PCR conditions and sample nucleic acid diversity (Nagai et al., 2022). Future research should focus on methods for confirming novel recombinants such as shorter, specific RT-PCR companion assays with low-frequency chimera production. Due to the difficulties discriminating PCR chimeras from genuine recombinants and in the absence of readily available reference material for mock community analysis to set PCR chimera filtering thresholds, it may be appropriate for metagenomic approaches to be used for the identification of novel recombinants.

### Noroviruses in Wastewater in England

The GI.2 genotype (GI.2[P2]) was detected in 100% of samples, this is in contrast to the Office for National Statistics’ (ONS) clinical data for the same period which stated GI.3 and GI.6 as the most frequently detected genotypes (3%) across the year; no p-type data were available (Office for National Statistics, 2023). This discrepancy may be explained by the absence of GI.2 in clinical cases due to its predominantly asymptomatic infections. GI.6 (GI.6[P11] and GI.6[P6]) were found in 60% of the samples while GI.3 types (GI.3[P3], GI.3[P10] and GI.3[P13]) present in 30% supporting the clinical data (Fig. 6). Our methods, however, also detected GI.9, GI.4 and GI.5 in over 40% of the samples indicating that clinical data may underestimate NoV diversity.

Greater congruence with the ONS data was seen for GII with three GII-types (GII.2[P16], GII.3[P12] and GII.4[P31]) detected in 100% of the samples and GII.4[P31] having the highest proportion of reads in all cases. The ONS reported 48% of cases were GII.4, 13% were GII.3 and 10% were GII.2 (Office for National Statistics, 2023) (Fig. 7). Without further validation, however, using these data in a quantitative fashion should be done with caution.

Comparing these two data sets is problematic as the ONS data are a summary of clinical cases in England across the year whereas our data are a snapshot of NoV diversity across England over several days. Differences between the two data sets, especially for GI, may also be due to the low case numbers reported (45) compared to GII (514) (Office for National Statistics, 2023). Furthermore, the samples used in this study were pooled, and any localised outbreaks could be diluted out of detectable range. Time-course studies with greater geospatial separation need to be conducted to assess the link between WBE and clinical data.

## Conclusions

We have developed a long amplicon nanopore sequencing method that allows the simultaneous dual-typing of NoV GI and GII in wastewater. RT, DNA polymerase, PCR conditions and library-pooling were optimised and a consensus-based bioinformatics pipeline was developed. We have shown that failure to optimise assays for WBE is likely to lead to underestimation of taxa-richness through loss of data to NSA. Furthermore, the appropriate bioinformatic interventions are required to prevent reporting of data from PCR chimeras.

Partial qualitative validation was performed with 88.9 and 78.6% of GI genotypes and p-types and 15.4 and 10.8% of the GII genotypes and p-types detected. Further validation is recommended before adoption at scale. Initial analysis indicated that our data matched the GII clinical observations although comparisons were difficult due to the nature of the data and lack of p-type in the clinical data. Future studies should focus on building a collection of NoV reference material and NoV negative wastewater to allow for full assay validation and optimisation of PCR methods to minimise chimeras. The development of methods for confirming novel NoV recombinants should also be prioritised.

Deploying this technique into a WBE scheme paired with studies into NoV shedding rates and virulence would allow better understanding of NoV diversity and prevalence within the population, outbreak tracking, genetic diversity and the importance of recombination in NoV evolution. Improved understanding of the biology and epidemiology of NoVs could lead to improvements in disease prevention and management and predictions of economic burden.

## Supplementary Information

Below is the link to the electronic supplementary material.

Supplementary file1 (PDF 590 KB)

Supplementary file2 (PDF 419 KB)

## Declarations

### Data Accessibility

Raw sequencing data comparing the wastewater optimised and unoptimised methods has been deposited to the National Center for Biotechnology Information databases under BioProject PRJNA1087315. Raw nanopore data and sanger sequences from the norovirus positive human faecal material is available at the BioProject and GenBank under the accessions PP542636, PP542646 and PP542648. The methods developed in this study are available in Online Resource 2 or on protocols.io 10.17504/protocols.io.8epv5xpmjg1b/v2.
