# Supplementary material for: Long Amplicon Nanopore Sequencing for Dual-Typing RdRp and VP1 Genes of Norovirus Genogroups I and II in Wastewater
Source: Food Environ Virol. 2024 Sep 6;16(4):479–91. doi: 10.1007/s12560-024-09611-5 (PMC11525389; doi:10.1007/s12560-024-09611-5)
Supplement: Supplementary file 1 — Supplementary file1 (PDF 590 KB) [file 12560_2024_9611_MOESM1_ESM.pdf]

Long Amplicon Nanopore Sequencing for Dual-Typing *RdRp* and *VP1* Genes of Norovirus Genogroups I and II in Wastewater: Online Resource 1

Scott, G.[1], Ryder, D.[1], Buckley, M.[1], Hill, R.[1], Treagus, S.[1,2], Stapleton, T.[1], Walker, D. I.[1], Lowther, J.[1] & Batista, F. M.[1]

[1] Centre for Environment, Fisheries and Aquaculture Science, Weymouth, United Kingdom

[2] UKHSA, Manor Farm Road, Porton Down, Salisbury, Wiltshire, SP4 0JG

Corresponding author: george.scott@cefas.gov.uk

This online resource contains the supplementary figures, equations and tables along with any additional references.

Contents

1.0 Supplementary Figures 2
2.0 Supplementary Equations 4
3.0 Supplementary Tables 5
4.0 References 11

1.0 Supplementary Figures

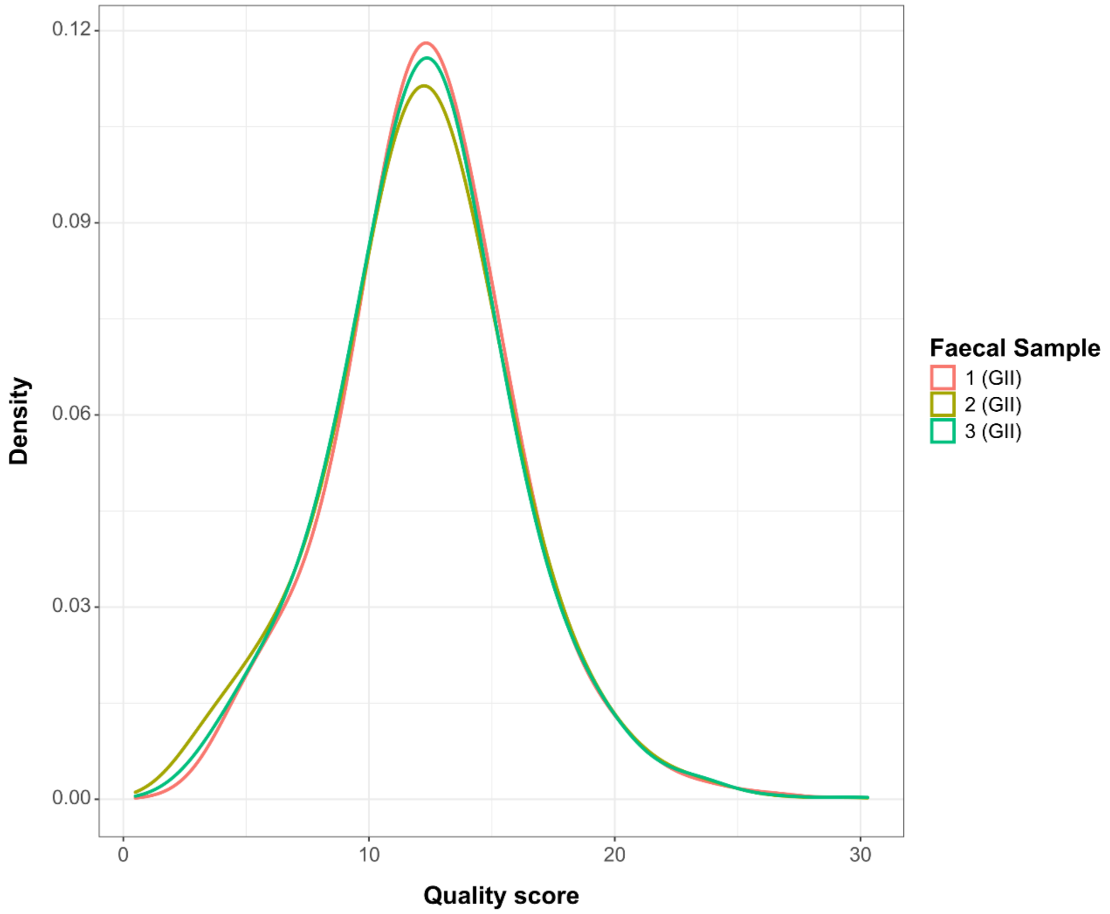

Supplementary Fig. 1 *The estimated quality of reads in three faecal samples assumed be associated with single-type infections.*

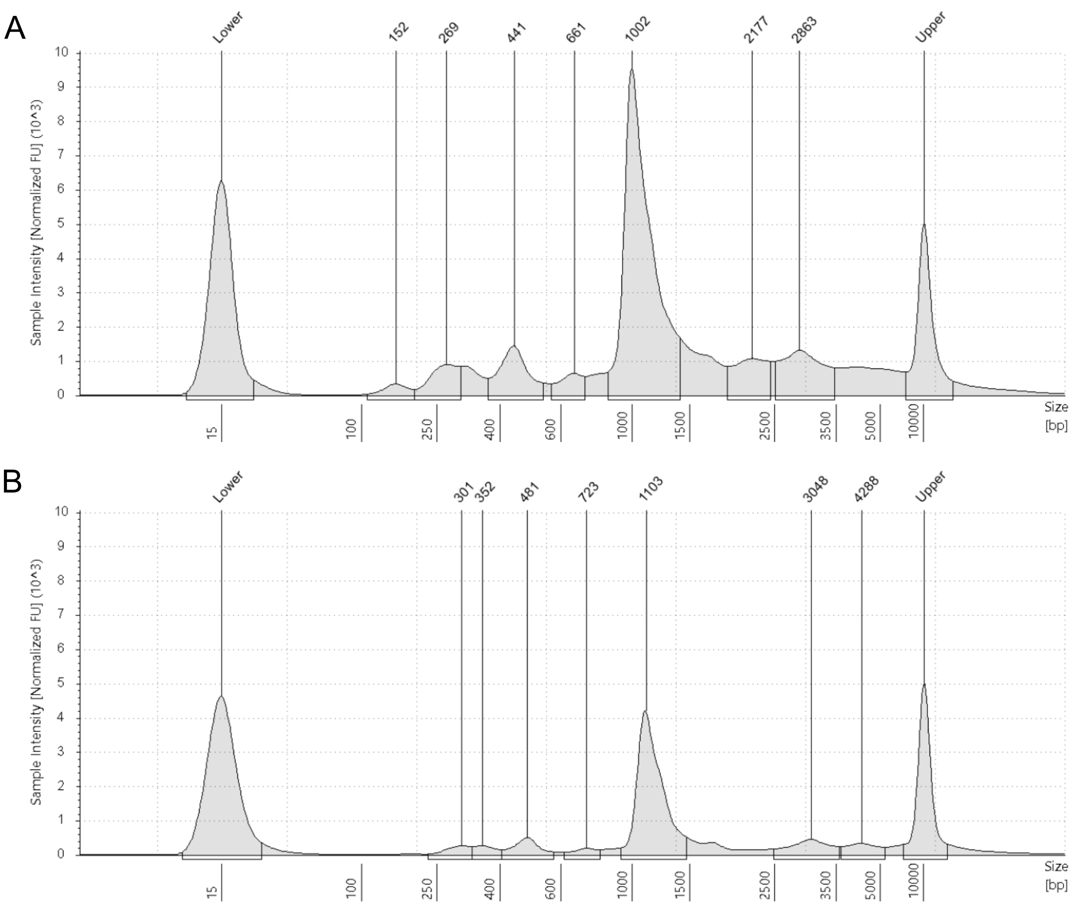

Supplementary Fig. 2 *TapeStation electropherograms of norovirus GI PCR before (**A**) and after (**B**) size selection.*

2.0 Supplementary Equations

$$P = 100\frac{m_a}{\sum_{i=1}^{n} m}$$

Supplementary Equation 1 *Calculating the percentage the amplicon of interest (P) within the total amplicon pool where m_a is the molarity of the amplicon of interest and m is the molarity of the different PCR products within a given sample.*

$$R = \frac{P_{GI}}{P_{GII}}$$

Supplementary Equation 2 *The ratio of the percentage of the amplicon of interest (R) between the norovirus genogroups I and II PCRs. P is as calculated in Equation 1 for genogroups I and II.*

$$GI\ moles = \frac{200}{R+1}$$

Supplementary Equation 3 *The moles of GI PCR product in fmol (GI moles) to be input into end-prep where R is the ratio of GI and GII amplicons as calculated in Equation 2.*

$$GII\ moles = R\frac{200}{R+1}$$

Supplementary Equation 4 *The moles of GII PCR product in fmol (GII moles) to be input into end-prep where R is the ratio of GI and GII amplicons as calculated in Equation 2.*

$$p = \frac{L_m - L_o}{L_o}$$

$$Q = -10\,log_{10}\,p$$

Supplementary Equation 5 *Estimating the probability that a base call is incorrect (p) and the overall quality of reads (Q) using the number of matching bases (L_m) and the overall length of the alignment (L_o) reported by minimap2.*

3.0 Supplementary Tables

Supplementary Table 1 *The pooled samples used in this study, their associated regions and the number of nucleic acid extracts in each composite.*

Sample	Regions	No. of samples
1	East and West Midlands	34
2	East of England	29
3	North West	28
4	South East	14
5	South West	14
6	Yorkshire	24
7	South East	14
8	South East	21
9	South West	14
10	South West	17

Supplementary Table 2 *Bioinformatic procedures, tools and commands.*

Procedure	Tool	Parameters	Reference
Basecalling and de-multiplexing	Guppy 6.3.9	Flow cell type 'FLO-MIN114' Kit type 'SQK-NBD114-96'	Oxford Nanopore Technologies, 2024
Read splitting	duplex_tools 0.2.14	--allow_multiple_splits Native	Oxford Nanopore Technologies, 2022
Read trimming	cutadapt 3.4	--action=trim -n 1 --discard-untrimmed -e 0.30 -O 12 –revcomp -g file:primer_sequences.fasta	Martin, 2011
Size filtering	SeqTK 1.3	seq -L 800	Li, 2018
Random sampling	SeqTK 1.3	sample 90000	Li, 2018
Identifying read overlaps	Minimap 2.24	-k19 -Xw19 -e0 -m100 -r100 -I 30M --cap-kalloc=8000m --cap-sw-mem=100m	Heng Li, 2018
Identification of chimeras and sequences with poor support	yacrd 1.0.0	-c 10 -n 0.2	Marijon, Chikhi and Varré, 2020
Read clustering and consensus sequence generation	NGSpeciesID 0.1.3	--q 15 --ont --consensus --max_seqs_for_consensus 10000 --rc_identity_threshold 0.9 --aligned_threshold 0.8 --mapped_threshold 0.8 --m 1100 --s 200	Sahlin, Lim and Prost, 2021
Indexing of consensus sequences	kma 1.4.9	-NI	Clausen, Aarestrup and Lund, 2018
Alignment, variant calling, and masking of poorly supported regions	kma 1.4.9	-vcf 1 -sam -ConClave 2 -bcNano -bc 0.7 -bcd 100 -t 20 -md 100 -1t1 -ont -ml 900 -xl 1100 -ef -mrs 0.92 -mrc 0.90	Clausen, Aarestrup and Lund, 2018
Rename consensus sequences	SeqKit 2.3.0	rename sequences.fasta OTU_	Shen *et al.*, 2016
Indexing of consensus sequences	Samtools 1.13	-faidx	Danecek *et al.*, 2021
Tabulating regions which were identified and masked by kma	SeqKit 2.3.0	locate --bed -P -r -p '^[agct]+' -p '[agct]+$'	Shen *et al.*, 2016
Sorting and complementing coordinates, and using the result to trim seqs	Bedtools 2.30.0	The sort, complement and getfasta commands were applied using output from the SeqKit and Samtools command.	Quinlan and Hall, 2010
Clustering of consensus sequences	CD-HIT 4.8.1	cd-hit-est -G 0 -c 0.95 -n 10 -d 0 -M 100000 -T 12 -g 1 -aL 0.9	Fu *et al.*, 2012
Removal of PCR chimeras	USEARCH 11	-uchime3_denovo -chimeras	Edgar, 2016

Supplementary Table 3 Nanopore sequence validation against Sanger sequencing.

Faecal Sample	Genogroup	Genotype and Subtype	Nanopore length (bp)	Sanger length (bp)	Q30 Sanger length (bp)	Indels	Nucleotide similarity (%)
1	GII	GII.4 New Orleans [P4] New Orleans	1008	945	862	0	100
2	GII	GII.4 Sydney [P31]	977	926	875	0	100
3	GII	GII.4 Sydney [P31]	926	951	884	0	100

Supplementary Table 4 *Novel norovirus GI types detected which may be PCR chimeras.*

Type	Sample	Read proportion (%)	Genotype parent read proportion (%)	P-Type parent read proportion (%)
GI.1[P2]	5	0.4	0	33
GI.2[P11]	2	0.8	52	27
GI.2[P11]	7	2.5	47	17
GI.2[P12]	8	1.1	57	23
GI.2[P1]	5	0.4	33	0
GI.2[P3]	7	0.4	47	22
GI.2[P4]	2	1.2	52	36
GI.2[P4]	6	0.7	18	67
GI.2[P4]	7	0.4	47	8
GI.4[P11]	4	0.8	7	9
GI.4[P2]	2	0.8	36	52
GI.6[P2]	7	1.0	17	47
GI.6[P3]	7	0.6	17	22
GI.6[P4]	3	0.9	21	8
GI.6[P4]	4	0.6	9	7
GI.6[P4]	7	0.4	17	8
GI.9[P3]	5	0.6	20	46
GI.9[P7]	4	1.0	57	19

Supplementary Table 5 *Norovirus GI genotypes detected.*

Genotype	Detected
GI.1	Yes
GI.2	Yes
GI.3	Yes
GI.4	Yes
GI.5	Yes
GI.6	Yes
GI.7	Yes
GI.8	No
GI.9	Yes

Supplementary Table 6 *Norovirus GI polymerase types detected.*

Polymerase type	Detected
GI.P1	Yes
GI.P2	Yes
GI.P3	Yes
GI.P4	Yes
GI.P5	No
GI.P6	Yes
GI.P7	Yes
GI.P8	No
GI.P9	Yes
GI.P10	Yes
GI.P11	Yes
GI.P12	Yes
GI.P13	Yes
GI.P14	No

Supplementary Table 7 *Norovirus GII genotypes detected.*

Genotype	Detected
GII.1	No
GII.2	Yes
GII.3	Yes
GII.4	Yes
GII.5	No
GII.6	No
GII.7	No
GII.8	No
GII.9	No
GII.10	No
GII.11	No
GII.12	No
GII.13	No
GII.14	No
GII.16	No
GII.17	Yes
GII.18	No
GII.19	No
GII.20	No
GII.21	No
GII.22	No
GII.23	No
GII.24	No
GII.25	No
GII.26	No
GII.27	No

Supplementary Table 8 *Norovirus GII polymerase types detected.*

Polymerase type	Detected
GII.P1	No
GII.P2	No
GII.P3	No
GII.P4	No
GII.P5	No
GII.P6	No
GII.P7	No
GII.P8	No
GII.P11	No
GII.P12	Yes
GII.P13	No
GII.P15	No
GII.P16	Yes
GII.P17	Yes
GII.P18	No
GII.P20	No
GII.P21	No
GII.P22	No
GII.P23	No
GII.P24	No
GII.P25	No
GII.P26	No
GII.P27	No
GII.P28	No
GII.P29	No
GII.P30	No
GII.P31	Yes
GII.P32	No
GII.P33	No
GII.P34	No
GII.P35	No
GII.P36	No
GII.P37	No
GII.P38	No
GII.P39	No
GII.P40	No
GII.P41	No

Supplementary Table 9 Trimming statistics from the nanopore sequencing run

Sample	PCR	Prior to trimming	After trimming	Aligned
1	Optimised	189871	115837	25478
1	Ta=50	343517	178910	35109
2	Optimised	296867	186637	66655
2	Ta=50	269260	151823	53328
3	Optimised	101838	72567	28005
3	Ta=50	228617	138937	39936
4	Optimised	211179	141071	46934
4	Ta=50	244136	137252	41135
5	Optimised	176652	120018	50043
5	Ta=50	202231	114026	34378
6	Optimised	127933	77030	33453
6	Ta=50	160346	76639	29239
7	Optimised	239487	153016	53502
7	Ta=50	215035	97940	16830
8	Optimised	127411	82976	27813
8	Ta=50	218580	127103	42306
9	Optimised	118572	77795	35158
9	Ta=50	314565	181594	45298
10	Optimised	80460	50634	22486
10	Ta=50	193210	99241	36497
Replicate 1 process control	Optimised	1463	484	0
Replicate 1 process control	Ta=50	1150	460	0
Replicate 2 process control	Optimised	1391	422	0
Replicate 2 process control	Ta=50	901	393	0
Replicate 3 process control	Optimised	837	325	0
Replicate 3 process control	Ta=50	1558	457	0
GI PCR 1 control	Optimised	936	313	0
GI PCR 1 control	Ta=50	1042	411	0
GI PCR 2 control	Optimised	1456	482	0
GI PCR 2 control	Ta=50	1145	434	0
GII PCR 1 control	Optimised	742	260	0
GII PCR 1 control	Ta=50	1403	545	0
GII PCR 2 control	Optimised	878	318	0
GII PCR 2 control	Ta=50	1321	523	0

4.0 References

Clausen, P. T. L. C., Aarestrup, F. M. and Lund, O. (2018) 'Rapid and precise alignment of raw reads against redundant databases with KMA', *BMC Bioinformatics*, 19(1). doi: 10.1186/s12859-018-2336-6.

Danecek, P. *et al.* (2021) 'Twelve years of SAMtools and BCFtools', *GigaScience*, 10(2), pp. 1–4. doi: 10.1093/gigascience/giab008.

Edgar, R. C. (2016) 'UCHIME2: improved chimera prediction for amplicon sequencing', *bioRxiv*, p. 074252. Available at: https://www.biorxiv.org/content/10.1101/074252v1%0Ahttps://www.biorxiv.org/content/10.1101/074252v1.abstract.

Fu, L. *et al.* (2012) 'CD-HIT: Accelerated for clustering the next-generation sequencing data', *Bioinformatics*, 28(23), pp. 3150–3152. doi: 10.1093/bioinformatics/bts565.

Li, Heng (2018) 'Minimap2: Pairwise alignment for nucleotide sequences', *Bioinformatics*, 34(18), pp. 3094–3100. doi: 10.1093/bioinformatics/bty191.

Li, Hang (2018) 'SeqTK: Toolkit for processing sequences in FASTA/Q formats'. Available at: https://github.com/lh3/seqtk.

Marijon, P., Chikhi, R. and Varré, J. S. (2020) 'Yacrd and fpa: Upstream tools for long-read genome assembly', *Bioinformatics*, 36(12), pp. 3894–3896. doi: 10.1093/bioinformatics/btaa262.

Oxford Nanopore Technologies (2022) 'Duplex-tools: Splitting of sequence reads by internal adapter sequence search'. Available at: https://github.com/nanoporetech/duplex-tools.

Oxford Nanopore Technologies (2024) *Guppy: Local accelerated basecalling for Nanopore data*, *Software Downloads*. Available at: https://community.nanoporetech.com/downloads.

Quinlan, A. R. and Hall, I. M. (2010) 'BEDTools: A flexible suite of utilities for comparing genomic features', *Bioinformatics*, 26(6), pp. 841–842. doi: 10.1093/bioinformatics/btq033.

Sahlin, K., Lim, M. C. W. and Prost, S. (2021) 'NGSpeciesID: DNA barcode and amplicon consensus generation from long-read sequencing data', *Ecology and Evolution*, 11(3), pp. 1392–1398. doi: 10.1002/ece3.7146.

Shen, W. *et al.* (2016) 'SeqKit: A cross-platform and ultrafast toolkit for FASTA/Q file manipulation', *PLoS ONE*, 11(10), pp. 1–10. doi: 10.1371/journal.pone.0163962.
